# As-Built Inventory and Deformation Analysis of a High Rockfill Dam under Construction with Terrestrial Laser Scanning

**DOI:** 10.3390/s22020521

**Published:** 2022-01-11

**Authors:** Peiwei Xiao, Ran Zhao, Duohui Li, Zhaogao Zeng, Shunchao Qi, Xingguo Yang

**Affiliations:** 1 State Key Laboratory of Hydraulics and Mountain River Engineering, Sichuan University, Chengdu 610065, China; 2019323060051@stu.scu.edu.cn (P.X.); 2021223060080@stu.scu.edu.cn (D.L.); 89022251@163.com (X.Y.); 2 Powerchina Sinohydro Bureau 7 Co., Ltd., Chengdu 610213, China; zr1625155@163.com (R.Z.); zengzhaogao@163.com (Z.Z.); 3 Department of Civil and Environmental Engineering, Carleton University, Ottawa, ON K1S 5B6, Canada

**Keywords:** terrestrial laser scanning, as-built dam model, dam deformation analysis, point clouds, concrete face rockfill dam

## Abstract

The construction of large earth/rock fill dams, albeit its remarkable progress, still relies largely on past experiences. Therefore, a comprehensive yet dependable monitoring program is particularly beneficial for guiding the practice. However, conventional measurements can only produce limited discrete data. This paper exploits the potential of the terrestrial laser scanning (TLS) for an accurate inventory of as-built states of a concrete-faced rockfill dam under construction and for a full-field analysis of the 3D deformation pattern over its upstream face. For the former, a well-designed 3D geodetic system, with a particular consideration of the topography, promises a regulated acquisition of high-quality and blind-zone-free point cloud at field and also eases the cumbersome data registration process while maintaining its precision in house. For the latter, a problem-tailored processing pipeline is proposed for deformation extraction. Its core idea is to achieve a highly precise alignment of the point clouds with Iterative Closed Point algorithms from different epochs in datum areas that displays a featured, undeformed geometry at stable positions across epochs. Then, the alignment transformation matrix is applied to the point clouds of respective upstream face for each epoch, followed by pairwise comparisons of multiple adjusted point clouds for deformation evaluation. A processing pipeline is used to exploit the peal scene data redundancy of the GLQ dam acquired at six different epochs. Statistical analysis shows that satisfactory accuracy for deformation detection can be repeatably achieved, regardless of the scanner’s positioning uncertainties. The obtained 3D deformation patterns are characterised by three different zones: practically undeformed, outward and inward deformed zones. Their evolutions comply well with real construction stages and unique 3D valley topography. Abundant deformation results highlight the potential of TLS combined with the proposed data processing pipeline for cost-efficient monitoring of huge infrastructures compared to conventional labor-intense measurements.

## 1. Introduction

The concept of building earth dams date back to the Third millennium BC when the natural soils/rocks were simply stacked together for flooding control and water supply. Despite the fact that no modern advanced theories could be utilised to assist their constructions and maintenances, some of the ancient dams are still surprisingly operating well. For example, the Jordan’s Jawa Dam built about BC. 3000 consisted of a slim stone wall for resisting erosion at upstream side and a dumpy rockfill for reinforcing stability at upstream side. This composite structure (5 m high) appeared to be impregnable such that mechanical intervention had to be employed to destroy it recently for other purposes [1]. This demonstrates the connatural stability feature of this reinforced design. This primitive design has been largely tapped by California miners in 1850s when the rock blasting-extruding riprap technique increased dam height to about 70 m. Since 1965, the advent of the vibratory-roller-compaction technique has further promoted the scales of the rockfills with heights of more than 300 m [2,3], and this composite design has evolved into the so-called Concrete Faced Rockfill Dam (CFRD) nowadays. Therefore, the prevailing CFRD is, in fact, the product of reinventing ancient wisdoms conceived from the Jawa Dam.

However, despite the great success of extra-high CFRDs, advanced techniques based on modern science have still not been rooted out, e.g., computer-aided numerical analyses and large-scale model test. In the former, current constitutive models are incapable of representing complete behaviours of rockfills and their highly variable characteristics [4,5,6,7,8]. While in the latter, real conditions could never be fully modeled due to the inherent size effect and distorted boundary conditions [9]. Therefore, the practices of higher and higher CFRDs still relies predominantly on past experience, engineering judgement and empirical methods [10]. In this regard, real field data on the dam performances serves as the most important information that contributes the progress of high CFRDs practice. They can be applied, for example, to (1) verification of the numerical analysis methods and model test protocols; (2) refinement of the empirical approaches with increasing volume of dataset; (3) improvement of the early warning signals for any catastrophic collapse and local incidents in existing dams; and (4) optimization of the field practices for dams under construction. Unlike other infrastructures, a dam constructed at a specific site usually behaves uniquely, characterised by its own materials and construction environments. Thus, data gathered at early construction stages of a dam, instead of from other dams, are most reliable for guiding the practices in its later stages. This strategy is particularly useful for construction of high dams (which usually takes several years) but has not been well implemented so far. One reason might be due to the incapacity of commonly established instrumentation system to support this strategy.

Current monitoring instrumentations can be categorized into two main classes by their data types, i.e., flow and deformation data. Flow monitoring will become a major concern during the operation phase if excessive leakage occurs due to severe cracking of the concrete’s face slab. Concrete face slab (CFS) cracking, indeed, has been observed in several well-known high dams, e.g., the 178-m high TSQ1 dam [11], 202-m high Campos Novos dam [12] and 140-m high Anchicaya dam [13]. Cracking development displays strongly random characteristics in space and has, thus, become one of the most imminent challenges in CFRD practice. Its randomness is closely linked to the 3D, differential deformation pattern of the underlying dam body near the upstream face. Therefore, fully monitoring 3D deformation patterns over the upstream face and its evolution, during the construction stage of CFRDs, can be very useful for understanding cracking behaviours in subsequent stages.

However, conventional near-surface deformation sensors (e.g., the hydraulic cells, extensometers, inclinometers, and leveling marks) cannot satisfy the above requirement. First, these conventional sensors are usually placed in several discrete points that are too limited to be interpolated for fully describing the dam’s random deformation feature. Second, they must be installed on the dam surface, and any construction interferences could affect their accuracy and functionality. Lastly, there is a lack of as-built inventory of construction history of the dam connecting different sensors in a complete framework; thus, it is difficult to interpret the relationships between readings at different locations. 

Recent digital advancements have resulted in several new displacement monitoring equipment that could overcome the limits of conventional instrumentations, e.g., photogrammetry, particle image velocimetry (PIV), unmanned aerial vehicle (UAV) and terrestrial laser scanning (TLS). With these techniques, it is possible to acquire high-resolution data points over a large area of the object surface in a very short time period and without direct contact with the object from which a global displacement map over the field of view could be obtained by an effective data processing algorithm for point clouds. In terms of applicability, photogrammetry and PIV are more applicable for close-range, small-scale measurements (e.g., soil elemental test [14]), while a UAV is adept in long-range, regional-scaled displacement measurements [15]. Comparatively, the TLS, with sufficient accuracy for middle-range, middle-scale measurements, appears to be mostly suited for monitoring the dam. In the past decade, several investigators have illustrated the applicability of TLS for deformation detection of concrete arch-dams (e.g., [16,17,18,19,20,21,22,23]). A few works have been conducted on the application of TLS for monitoring earth/rockfill dams. Berberan et al. [24] have detected noticeable crest settlements of Lapão earth dam (39.5-m high) during its first reservoir filling from TLS data. The authors and his co-workers obtained the post-construction deformation behaviour of a clay-core rockfill dam (240-m high) with the TLS technique [25]. 

In summary, the current practice of higher and higher CFRDs are facing several challenges, e.g., alleviating face slab cracking that presents random and unpredictable characteristics, and is closely linked to the deformation behaviour of the underlying dam body. Due to the limitations of theoretical and experimental techniques, a well-designed field monitoring scheme, which is capable of recording a complete picture of the dam’s behaviour at its different construction stages, can offer a valuable database for investigating the fundamental behaviour of large volume rockfills and for guiding construction practices on site. This requires that a large volume of high-accurate field data to be acquired in a prompt manner, which cannot be realized by any conventional sensors. In this regard, the promising potential of the TLS for measuring dam behaviour has only been slightly touched so far, and the formal protocols for the instrument field operations and data postprocessing strategies have yet been established.

In this paper, we presented a well-designed application of TLS for monitoring the GuoLangQiao (GLQ) Dam (a 183.6 m high CFRD) that is under construction. Based on the established 3D geodetic system, the digital as-built dam models at several different construction stages have successfully been re-constructed from high-accuracy, high-intensity point clouds acquired in different epochs. Peal scene data redundancy has been exploited to detect the full-field 3D deformation pattern of the upstream face and its evolution by a proposed processing pipeline. The obtained results comply well with real construction stages and the unique 3D valley topography. Various important aspects, related to the machinery specifications in the field and data postprocessing strategies for the unprecedented size of points, have been discussed in detail. The work presented in this paper can serve as a good reference for the application of TLS to measurements of the as-built behaviour of the CFRDs. Moreover, for the first time, the great potential of the TLS technique for understanding 3D deformation patterns of CFRD during construction stages is also shown, and this includes their possible implications for cracking behaviour of concrete face slabs.

## 2. Project Overview

**Figure 1:**
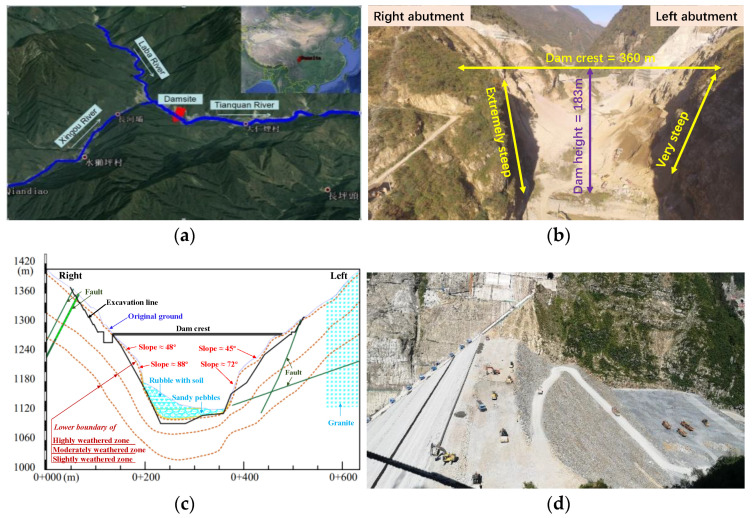
Project site: (**a**) dam site in the map; (**b**) a picture of the project site taken before construction commencement; (**c**) a sketch of cross-section of the site valley; and (**d**) a photo taken in September 2021.

Sanctioned in 2011 under the Sichuan Provincial Development and Reform Commission, the GLQ Hydropower Project is the first key flagship reservoir project among the cascade hydropower stations planned in the Tianquan River basin, which flows through the City of Tianquan (37 km away from the dam site), Sichuan, China (Figure 1a). According to the design, the reservoir has will have a catchment area of about 2220 km^2^ with a total capacity of about 184 million m^3^. The normal storage level is 1280.00 m, and the dead water level is 1220.00 m. This GLQ project is designed as a hydroelectric power plant of seasonal regulation. Three hydraulic Turbine Generators will be installed with a total power generating capacity of 210 MW.

### 2.1. Site Topography and Geology

Site topography and geology are both important influencing factors on construction organization optimization and deformation patterns of the dam during the construction phase. Figure 1b shows a picture of the project site that was taken before the commencement of construction from a viewpoint. As observed, site topography can be characterized as a typical deep and narrow ‘V-shaped’ valley located in the transition zone from Tibetan plateau–Sichuan basin in western China. Figure 1c provides a sketch of the cross section of this valley. The valley’s base is located at elevations of 1122–1130 m, and its width varies from 20 to 40 m observed in dry seasons. The left abutment is relatively steep within slopes of 50–70°. The right abutment is much steeper with slopes of 80–90° (close to cliffs of 60 m high) in its lower portion and becomes gentler (slopes of 20–30°) above the elevation of 1134 m. The top elevations are 1900 m for the left abutment and 2500 m for the right with an elevation deference of >700 m. The dam will be constructed to the elevation of 1280.5 m, rising beyond the elevation where the right abutment slope changes. Therefore, it is expected that the constraints from this bilaterally asymmetric valley will have significant effects on the deformation behavior of the dam. 

The alluvial layer with thicknesses of 3–20 m is mainly located at the riverbed, several fan-shaped or conical sediments are accumulated at the slope’s toe (see Figure 1c). These alluvial materials consist primarily of gravels mixed with silty clay, with loose structure and poor gradation. At the two steep abutments, hard bedrocks are exposed as steep cliffs, mainly composed of fleshy red granite and blackish-grey diorite. The horizontal depths of strongly, weakly and slightly weathered zone are 2–12 m, 46–82 m and 105–195 m at the left and are 0–15 m, 49–102 m and 104–195 m at the right, respectively. These bedrocks are measured to have high densities, low water absorptions and high compressive moduli and strengths (ranging from 102.4 to 155.6 MPa depending on the weather conditions). No regional faults, except for several small-scale faults and dense rapture zones, are found near the dam site. With a proper reinforcement measure, this site could serve as a firm foundation for high dams.

### 2.2. Dam Design and Filling

**Figure 2:**
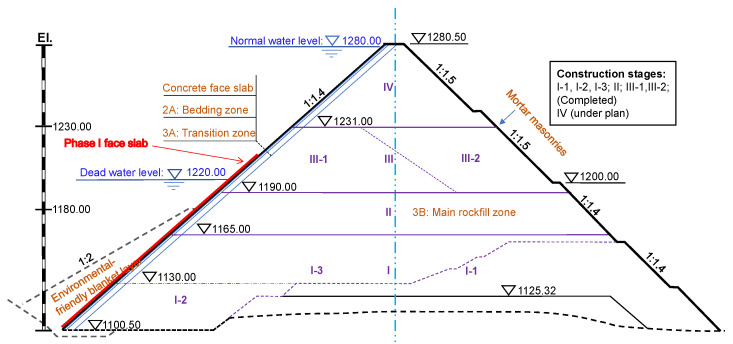
The maximum cross section of the GuoLangQiao dam.

The dam is designed as a typical CFRD with a maximum height of 186.30 m and crest length of 360.00 m. The upstream slope is uniform (1V:1.4H), while the downstream slope is designed to be slightly gentler (1V:1.5H) near the toe than that (1V:1.4H) near the crest. The structural composition of the dam is classical and comprises five zones with varying granulometry (Figure 2), namely, an environmentally friendly blanket layer (1A: Clay, 1B: Fly ash), the bedding zone (2A: Max. particle size of 100 mm) beneath an extrusion side wall, a transition layer (3A: Max. particle size: 300 mm) and the main rockfill zone (3B) covered by mortar masonries over the downstream face.

After removing riverbed alluvium, the rockfill deposition in successive layers of about 0.8 m thick commenced in September 2018. Local diorite and granite extracted from three quarries nearby have been processed for the main rockfill materials. Compaction quality was controlled as follows: dry density > 2.19 t/m^3^ for both types of rocks. Dam filling was expected to be completed at the end of 2021. However, the construction process had been unfortunately interrupted twice: the first one (from July to October 2019) was due to excavation-induced slope failures at one quarry, while the second one (from December 2019 to April 2020) was due to the COVID-19 pandemic. Thus, dam filling only reached the elevation of 1232 m in September 2021 (Figure 1d), which will be re-initiated in early 2022 after pouring the Phase-I concrete face. 

## 3. Field Work and As-Built Inventory

### 3.1. Instrument Settings

The 3D TLS technique can capture the geometry of an engineering object’s surface by an increasingly fast speed based on the recent advancements in various rangefinder techniques. Categorized by fundamental principles, there are three types of rangefinder techniques, namely, the time-of-flight, phase and triangulation-based measurement. Their detailed physics and most appropriate application scenarios can be found elsewhere (e.g., [26,27,28,29]). Among them, the long-range, time-of-flight-based technique appears to be most appropriate for dam engineering. For example, González-Aguilera et al. [17] utilized Trimble GX200 (0.35 km range) for detecting the response of the Las Cogotas arch dam of 66 m in height subjected to one cycle of reservoir’s water level change in Spain. Alba et al. [16] measured the spatial variation of the downstream face of the Cancano arch dam with 136 m in height in Italy by combining Riegl LMS-Z420i (1 km range) and Leica HDS 3000 (0.3 km range). Both scanners are based on the time-of-flight principle. 

Different from the concrete arch dam, GLQ CFRD has a flat body with a significantly larger volume and more gentle slope surfaces. Thus, the long-range scanner that could cover a wider field-of-view is certainly more advantageous. RIEGL VZ-2000i [30] adopted in this study allows measurements of distances of up to 2.5 km at 90% reflectively, while accuracy is tested to be 5 mm at 100-m distance by the vendor. The rangefinder works with a laser source with a near-infrared wavelength. The emitted laser pulse is deflected by an inner fast-rotating multi-facet mirror into multiple directions within a vertical plane. Meanwhile, the entire optical head mounted on a stationary pedestal rotates slowly around the vertical axis to sample the surrounding environment. Its field of view (horizontal 360° and vertical 100°) was fully exploited to capture as much surrounding information as possible to aid data post-processing. It is worth stressing that both measurement accuracy and maximum range are affected by various factors, including incidence angle, atmospheric visibility, dust obstacle and pulse repetition rate (PRR), etc. Considering that effective measurements could be reduced by unfavorable environments (e.g., construction smogs), a low PRR of 50 kHz was set to maximize the measurement range for capturing the massive dam body as much as possible from a single scan. The angular resolution was fixed to 50 millidegrees for all scans, although the actual sampled resolutions (i.e., the intervals between points on the object face) depend on scanner–object distance and incidence angle. By combining these parameters, a single scanning task from one point-of-view could be completed in 12 min, which produces around ten to thirty million points. This is a reasonable number of points that could be manipulated with a decent desktop. However, a single point-of-view cannot offer a full picture of rising dam morphology at any time in such narrow valley, which, in turn, requires a combination of multi-positional scans in an acquisition campaign in a well-established geodetic network. 

### 3.2. Geodetic Network

**Figure 3:**
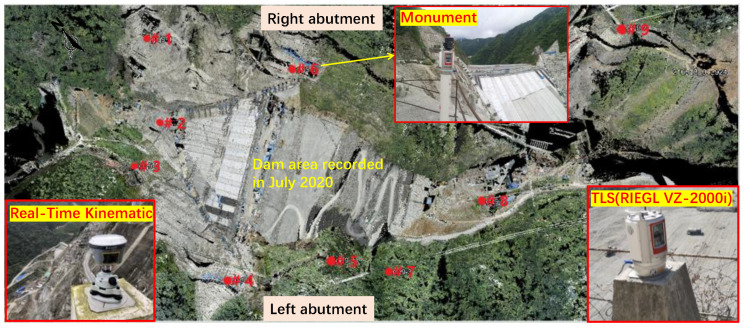
Established geodetic network consisting of nine permanent monuments that are used to support Real-Time Kinematics and Terrestrial Laser Scanner.

Considering specific site topography conditions, the established geodetic network consists of a total of nine permanent monuments (numbered as # in Figure 3), which serves as pedestals for the scanner during each measurement epoch. Among them, points 1# and 6# are located on the left abutment and points 4#, 5# and 7# are located on the right. These five points were placed with care such that they can ensure the following:

(a) A relatively complete view of the rising compaction face at the dam top can be obtained at any construction period;
(b) A sufficient area of overlap can be achieved from any two adjacent scans. This is essential for splicing neighboring point clouds gathered at the same epoch (i.e., co-registration);
(c) The point cloud from one scan can complement the other from the adjacent scan to yield sufficient data points in the overlapping area. This is because the point resolution in the overlaps from one scan might be largely reduced due to the high incidence angle;
(d) Orientation bias and occlusion could be minimized. Since laser pulses from one point of view might be hidden by local bulge areas in this valley;
(e) Monuments are stable and safe for personnel access, considering the local steep cliff topography (see Figure 1b).

Points 2# and 3# are located at the upstream area in order to detect the deformation characteristic of the extruded curb during construction period. The distances between points 2# and 3# and the extruded curb are 100–300 m and 80–350 m, respectively. On the other hand, points 8# and 9# are placed for observing the downstream slope. The combination of the nine points of view (see Figure 3) basically allows the reconstruction of a complete 3D as-built dam model once co-registration of different scans from the same epoch has been accomplished. The measurement of the geodetic network has been carried out by means of the Real-Time Kinematic (RTK) technique, which makes use of GPS signals to obtain absolute 3D coordinates of each vertex on the monument with a millimeter accuracy under static conditions. This geodetic system has dramatically facilitated the 3D model reconstruction process. The determined RTK coordinates provided a reference for validating the co-registration of different scans (which is primarily based on the overlapping areas between neighboring scans), and they also serve as data for georeferencing the point clouds from different epoch into a common coordinate system.

### 3.3. Scanning Epochs and Co-Registration/Georeferencing

A total of six TLS epochs have been undertaken as construction progressed. Measurements were originally scheduled more intensively but were interrupted by the slope failure at one quarry and the COVID-19 pandemic from October 2019 to April 2020. Each epoch included at least nine points of view at the permanent tripods of the geodetic network. Additional scanning station could be added if desired, which only marginally increases time cost (12 min for each scan). The entire process, including the transfer and installation of instruments, can be completed in under half a day by one trained personnel for each epoch round. 

**Figure 4:**
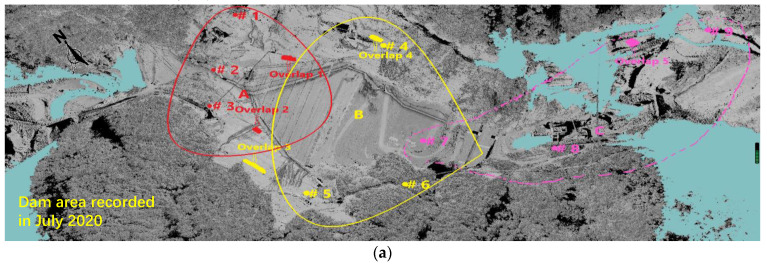

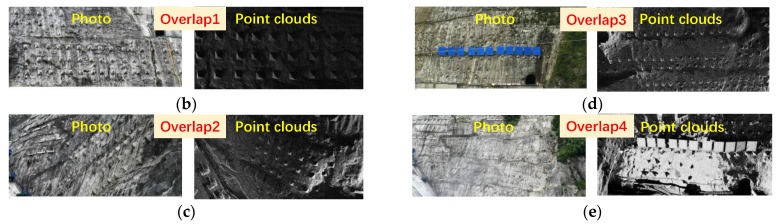
Two-stage co-registration strategy for creating a single registered TLS point cloud mosaic for epoch 2007 as an example: (**a**) three groups for the two-stage co-registration strategy; (**b**) overlap 1 for sub-registration; (**c**) overlap 2 for sub-registration; (**d**) overlap 3 for sub-registration; and (**e**) overlap 4 for sub-registration.

The raw point cloud downloaded from the scanner contains a considerable part of interferential noisy points, e.g., vegetation and various construction machineries. These unavailing points were first filtered out to ensure both quality and efficiency of subsequent co-registration and georeferencing. This was performed by a semi-automatic procedure. The surface of the dam under construction could be reasonably well described using an estimated ground terrain, then the off-terrain points (i.e., the vegetation and machinery) were removed. However, for overhangs, the reference planes must be specified manually at such local places in a separate manner, and the off-plane points were regarded as noisy data and then removed. This could be time consuming in some cases but is quite practical for establishing the 3D dam model, and the entire process for one epoch could be completed in less than one hour by trained personnel. It should be noted that co-registration cannot rely solely on 3D coordinates of the monuments, because any slight deviation from their actual values (which may arise from the RTK measurement inaccuracies and multi-epoch positional uncertainties) can result in notable registration errors. Thus, a two-stage co-registration strategy was proposed and applied to the filtered point clouds. The scans from different monuments were clustered into three groups (Figure 4). The first stage involved three sub-registrations of different scans within the same group (e.g., scans from points 1, 2 and 3). In the second stage, three grouped point clouds were co-registered to create a single registered TLS point cloud mosaic for each epoch. For example, the points for epoch 1903 (1903 represents March 2019: the first two numbers denote “Year” and the last two denotes “Month”, the same hereinafter) are shown in Figure 4a. For both stages, the iterative closest point (ICP) algorithm originally proposed in [31,32] was applied only to the selected adjacent overlapping areas that are stable and has unique geometric shape features to reduce the side effect of remaining noisy points. The ICP algorithm is the one of the most common techniques for data registration. The underlying idea of the ICP algorithm is to establish transformation matrices, for which its application to source point cloud (P) will result in the best matching of overlapping regions between source and reference ($P_{0}$) point clouds. The ICP algorithm attempts to solve an optimization problem where the cost function is defined as $C\left(R,T\right)=\Sigma \left(Rp^{i}+T-p_{0}^{i}\right)$, where $p_{0}^{i}$ and $p^{i}$ are the coordinates of the $i^{th}$ pair of the closest correspondent points from $P_{0}$ and P, respectively. R and T are the rotation and transformation matrices to be iteratively solved for minimizing the cost function. 

In this paper, the ICP algorithm embedded in the RiSCAN PRO software [30] was adopted, which found the optimal solution by a least square fitting technique. With the user friendly window, the entire ICP operation consists of three main steps: (1) establishment of good initial guesses for R and T. This was achieved by coarse registration by manually selecting several (theoretically ≥ 4) temporarily defined corresponding points. For our case, around ten points were selected over the overlapping area with well recognized features between any two data sets, and most of them were distributed over billboards and anchored covers on two abutments. The authors’ experience showed that if these points were distributed over a relatively large area and across a large elevation difference, a better initial guess could be obtained, yielding an error of around 0.2–0.3 m. The error is defined as the standard deviation of the distances between corresponding points, and the outlier points were not considered. (2) Activation of the optimization procedure: As described above, the closest point from the source to any one point from the reference point cloud must be found to evaluate the cost function. A search radius of around 2 times the error was prescribed to save the computational resource. Meanwhile, the *minimum change* error of 0.1 m was set as the termination criterion for the optimization of R and T during this step. Afterwards, a new error will be yielded. (3) Refinement of the optimization solution: It is usually impossible to obtain the solution with desired accuracy by one trial. Thus, the above optimization procedures usually required repetition. For each repetition, the search radius and minimum change of errors were set 2 times and one-tenth of the error value that was obtained during the previous optimization step. Generally, the error could be reduced to around 0.05 m by 4–5 times repetition and was not likely to decrease further with more repetitions. It should be noted that the above ICP operation was only applied to the selected overlapping areas (e.g., billboards and anchored areas on the two abutments; Figure 4b–e show pictures and corresponding point clouds) during each sub-step of the two-stage co-registration strategy mentioned above. After both stages, registration errors were quantified by mismatches of the relative coordinates of scanner’s positions among epochs after affine geometric transformation and by checking their deviations from respective RTK measurements. A refinement of co-registration at either step could be undertaken until errors were in the range of centimeter. Finally, these co-registered point cloud mosaics from all epochs were georeferenced relative to RTK measurements in the local topographic reference system with the least squares fit algorithm.

### 3.4. As-Built Dam Inventory

**Figure 5:**
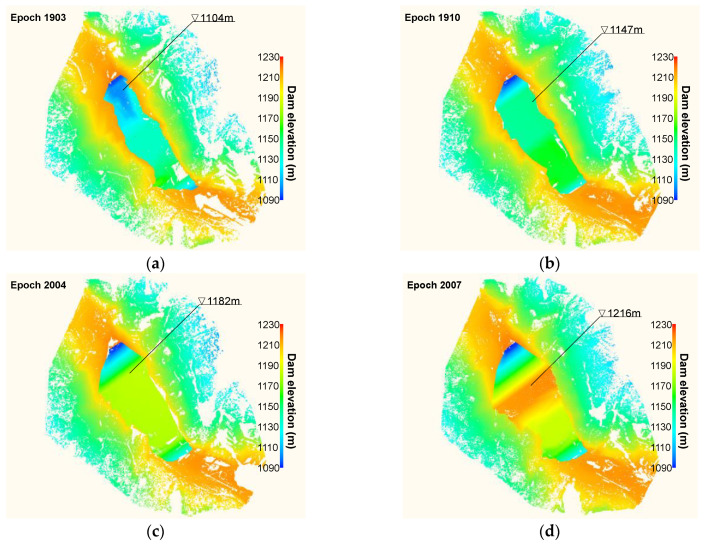

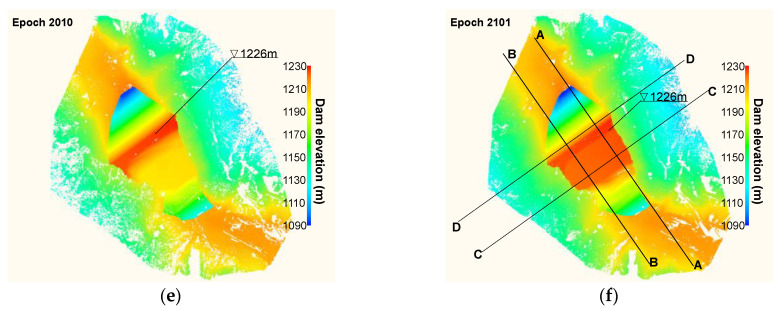
Six reconstructed point cloud 3D dam models for different epochs: (**a**) epoch 1903; (**b**) epoch 1910; (**c**) epoch 2004; (**d**) epoch 2007; (**e**) epoch 2010; and (**f**) epoch 2101.

As described, it becomes quite feasible to reconstruct the as-built 3D dam model from TLS point clouds in an efficient manner. The entire workflow at any selected construction stage, including field data acquisition and laboratory data processing, can be completed in under two days. Figure 5 illustrates six reconstructed 3D dam model for different epochs, which shows that the dam body grows as construction progresses. The reconstructed models that are digitally represented by point clouds offer comprehensive and highly accurate information for other specific applications. They could be incorporated into other information-intensive computer-aided tools (e.g., building information modelling) for optimizing subsequent construction planning, for instance, to increase the manpower and material resources’ usage efficiency based on more accurate, real-time excavation-fill balance calculations. They could also be used to reinspect construction quality by tracking back to built details from previous models.

Thus, the quality of the point cloud inventory model depends not only on point coordinate accuracy but also on its completeness (the occlusion area) and sampling resolution [33]. Sampling resolution can be quantitatively evaluated by density spatial distribution. Every single point can be assigned with a local density value that is defined as the quantity of points per unit of its neighboring area. The Kd-tree based search algorithm was adopted to find neighbors within a radius (*r* = 0.5 m) of each query point, and then its density was calculated as NoN/($\pi r^{2}$), where NoN represents the Number of Neighbors. The first and core step of the KD-tree (stands for k-dimensional tree) search algorithm was to build a binary search tree for storing k-dimensional points. For its application in point cloud analysis, the points in the 3D space were split recursively by a series of axis-aligned planes. Splitting planes were chosen to be perpendicular to x-axis, y-axis and z-axis in sequence and to pass through the median point of the remaining points with respect to their coordinates in the corresponding axis. For instance, a plane perpendicular to x-axis should be created by using the median (i.e., a non-leaf node) of x values of the coordinates, and all remaining points with smaller and larger x values represent left and right sub-trees, respectively. Once this space-partitioning data structure was constructed, the nearest neighbors of a given point can be effectively found by neglecting a large portion of the tree’s branches. First, the algorithm can quickly place the given point into the corresponding subspace (or the leaf node) at the last level of the KD-tree by assuming that this point was inserted into the point cloud, then the nearest point in this leaf is taken as the current best point. Then, a backtracking operation was performed to check if there were points in the other side under the current non-leaf node that are closer than the current best. If so, this entire branch in this side should be traversed; if not, the algorithm moves up to the parent non-leaf node. The backtracking process was repeated until root node was reached. In this study, the C++ based data processing algorithm from the Point Cloud Library [34], which is an open-source platform, is adopted to perform the KD-tree based search operation. Specifically, the generic type of 3D locator, KdTreeFLANN, was used, which includes two primary C++ objects, namely, *setInputCloud* and *radiusSearch*. The pseudo-code formulated to calculate density distribution over a point cloud is as follows.
**Calculate the density based on the KD-tree based search algorithm****Require:** Cloud: the input point cloud to be searched, e.g., the data for epoch 2007.
**Require:**
*r*: the search radius set to be 0.5 m in our study.
**Step 1:** kdtree.setInputCloud (Cloud); (establish the kdtree for the input cloud)
**Step 2:** for *i* = 1: N (N is the number of points in the input cloud)
kdtree.radiusSearch(Cloud(*i*), *r*, pointIdx, pointSquaredDistance)
density(*i*) = pointIdxRadiusSearch.size/($\pi r^{2}$)
**End**

**Figure 6:**
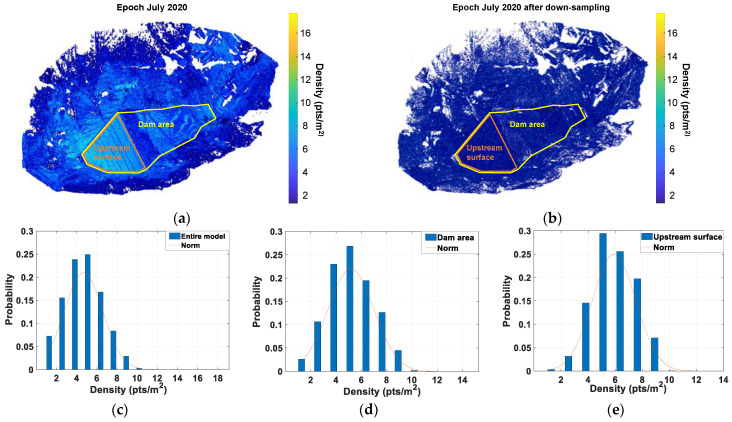
Point density distribution for epoch 2007: (**a**) calculated density mapped back to the original point cloud; (**b**) density distribution after down-sampling; (**c**) probability distribution of the density value for the entire model; (**d**) probability distribution of the density value over the dam area; and (**e**) probability distribution of the density value over the upstream surface.

First, the kdtree.setInputCloud object was used to establish the so-called KdTree for the input Cloud, e.g., the data for epoch 2007 in our study. Then, the kdtree.radiusSearch object was used to find all the neighboring points within a certain radius for every point in the input Cloud in the “for” loop. Meanwhile, the neighboring points’ indices (pointIdx) and their distances (pointSquaredDistance) to this query point within the specified radius can be returned, and the local density around this query point was calculated in the same loop. The “for” loop is terminated until density values were obtained for all the points in the input Cloud. Figure 6a shows the calculated point density distribution mapped back to the original point cloud for epoch 2007 in this manner. Figure 6c–e show its probability distribution over the entire model and dam area and upstream area, respectively. It is observed that the point densities over the majority of the areas range from 4 to 6 pts/m^2^. The density values over the dam areas range from 4 to 12 pts/m^2^, and the relatively high densities (8–12 pts/m^2^) are observed in the upstream surface, since it can be scanned from three points of view (i.e., points #1, #2 and #3, see Figure 4). Very high-density values (>14 pts/m^2^) are only observed in a few local areas. The relatively lower density values of 1.7–2 pts/m^2^ occur in the abutment mountain areas on both banks, where several inevitable occlusion areas are also observed. Overall, a satisfactory high-density distribution is obtained over the area of interest (dam area). This indicates the high quality reconstructed point cloud models. Rather than increasing the model quality, having too many high-intensity points in the local areas may slow down data postprocessing and require extra disk and memory storage. Thus, a down-sampling process based on the box gird filter was applied to the point clouds. Down-sampling operations were performed by using the Octree filter algorithm embedded in the software RiSCAN PRO [30]. First, the 3D space was divided by 3D cells for which their dimensions were set to be in all directions (0.1 m in all three directions). If the number of points in a cell is less than a specified minimum value (1 point), those points were removed. If a cell contains more points than a threshold (10%) resolution, they were merged to a single point by averaging their coordinates. This down-sampling operation generated point cloud models (see Figure 6b for epoch 2007) with density (about 1.5 pts/m^2^) uniformly distributed over space and without losing important local geometric details. 

**Figure 7:**
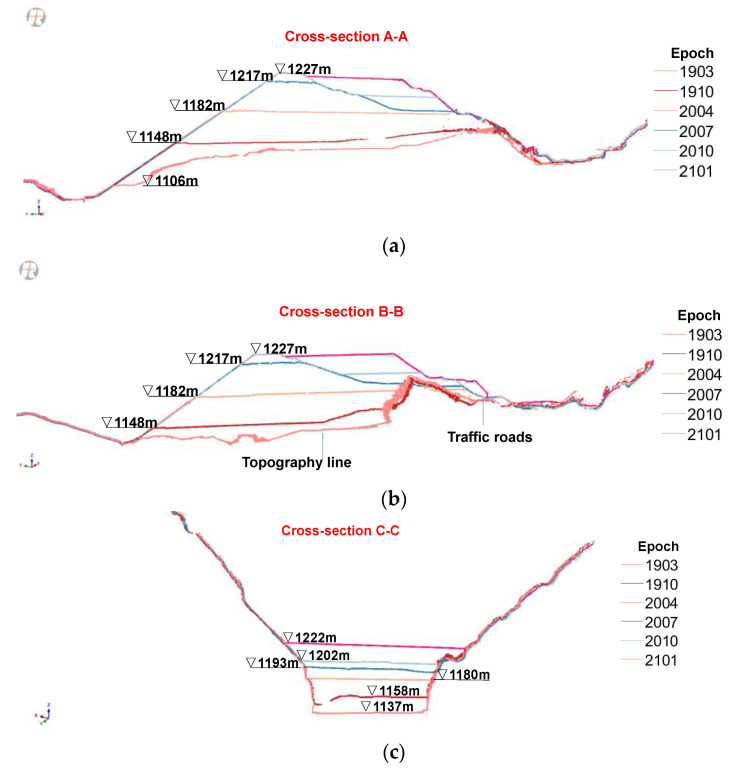

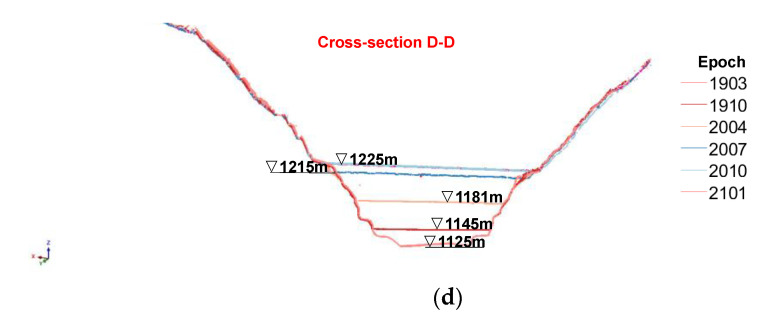
Four cross sections of point clouds showing the dam’s construction history: (**a**) cross-section A-A; (**b**) cross-section B-B; (**c**) cross-section C-C; and (**d**) cross-section D-D.

Finally, any 2D cross sections of point models could be selected, again by the Kd-tree based search algorithm, for a better picture of the dam’s construction history (Figure 7). Two longitudinal cross sections (along the flow direction) show the difference in the dam body at different stages between upstream and downstream portions (Figure 7a,b). The two transverse cross sections (perpendicular to the flow direction) show again the valley’s asymmetry (Figure 7c,d). The positions of the four cross-sections are marked in Figure 5f. The combination of these four cross sections (or any other) provides a detailed 3D geometric feature of the rising dam body from 2D perspectives. Moreover, different regions of dam surface can also be automatically segmented based on the evaluation and clustering of the points’ normals. The normal of any query point is defined as the normal of the local plane fitted to its neighboring points. The points on the upstream surface were segmented in this manner for a detailed analysis of its deformation behaviour during construction.

## 4. Deformation Analysis of the Upstream Face

A good understanding of the 3D spatial variation of the upstream face deformation characteristic could be extremely useful for guiding and optimizing subsequent engineering activities. For example, the deformation characteristic of the Phase-I face slab (see the red line in Figure 2) is closely linked to the deformation of its underlying dam body subjected to further dam filling, which could be predicted based on deformation trends revealed from previous point cloud inventories. Compared with traditional point-wise measurements, a more complete picture of the 3D deformation characteristic is reserved in TLS point cloud data redundancy, which, however, must be exploited by a wise method. 

### 4.1. Analysis Methodology

The deformation analysis of the TLS point clouds usually involves a detailed comparative and statistical investigation of the difference between point clouds of different epochs. The point cloud comparison methods in the literature can be categorised into three groups according to geometric information that is used to represent the objects scanned before and after deformation, namely, (a) the point-to-point-based method [35], (b) the point-to-surface-based method [36] and (c) the surface-to-surface-based method [37]. The first method calculates the magnitude of deformation as distances between a point from one epoch and its correspondence from the other. However, it is never possible to sample the same points on the object’s surface at multiple epochs due to a variety of reasons (e.g., shifting scanner positions, varying environmental conditions, changing laser beam width and deforming object shape). The second and third methods involve the transformation of one of or both point clouds into continuous surfaces with a suitable mathematical technique (e.g., the triangulation). One main advantage of mathematical transformation is that several potential errors or uncertainties (e.g., the systematic errors, reference frame instability and data noise associated with acquisition and alignment) could be largely alleviated during the surface generation process.

In this study, the “point-to-surface” based method is adopted to systematically estimate the deviation between point clouds. In order to extract spatial and temporal evolutions of the 3D deformation field of the upstream surface, a total of ten pairwise comparisons, divided into four groups, were carried out on point clouds of five different epochs (Table 1). In each group, one of the point clouds from the former four epochs was taken as reference data, and the point clouds acquired after the corresponding reference epochs were used as the test data. In each comparison, a Triangular Irregular Network (TIN) model was first generated by the classic Delaunay triangulation method to represent reference data, then the shortest distance from any one point in the test data to its nearest triangle plane in the reference TIN model was calculated as the displacement of that point between two epochs. In this manner, the direction of displacement vector varies from point to point and the obtained displacement field demonstrates a true 3D characteristic in space.

### 4.2. Accuarcy Examination

The key to successful displacement analysis is to ensure that all the point clouds from different epochs are geo-referenced in a common coordinate system with sufficiently high accuracy. This prerequisite is also one of the most challenging tasks due to two reasons. First, direct application of the ICP algorithm is paradoxical for deformation analysis, since its accuracy relies on the fact that two point clouds demonstrate identical geometric shape, while it is obviously not the case when deformation detection is of the primary concern. Second, several commonly used artificial targets that were measured by the Total Station might serve as a good auxiliary reference for geo-referencing; however, it becomes impractical to ensure their durability and functionality at this site of intensive engineering activities. 

**Figure 8:**
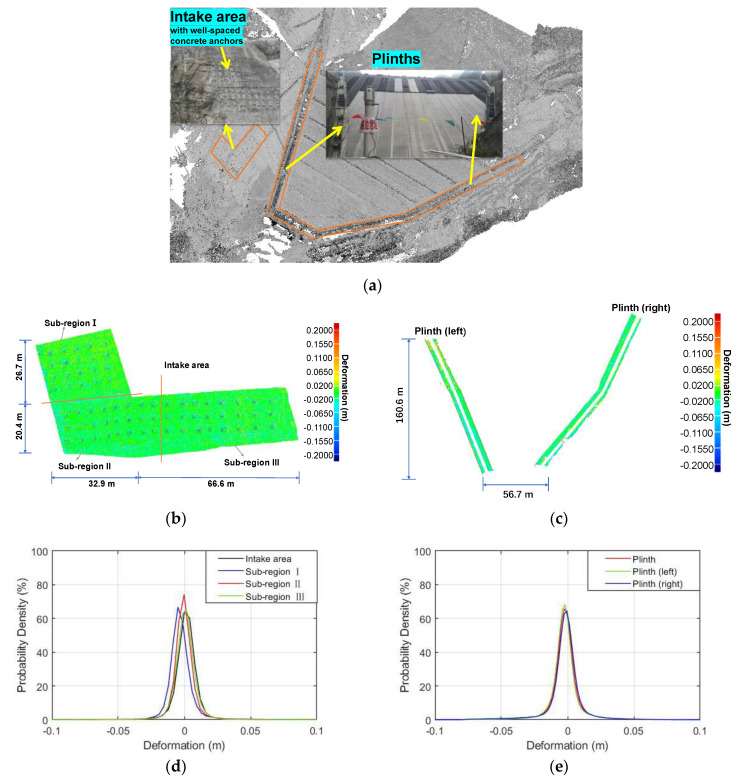
Distribution of nominal deformation (error) over datum regions between 1910 and 2007: (**a**) the stable datum regions for geo-referencing; (**b**) 3D nominal deformation for the datum region of the intake area; (**c**) 3D nominal deformation for the datum region of the Plinth; (**d**) probability density distribution of the nominal deformation for the datum region of the intake area; and (**e**) probability density distribution of the nominal deformation for the datum region of the Plinth.

Previous co-registrations showed that a high-accuracy outcome could be obtained if the ICP algorithm was applied only to the selected adjacent overlapping areas that has unique geometric shape features. This idea of using the ICP algorithm can be extended to geo-reference multi-epoch point clouds into the same framework if these selected areas remained stable and were not affected by environmental conditions during the construction period. A careful inspection revealed that several regions of point clouds display such unvaried features, namely, a region near the intake of the diversion tunnel on the right bank slope, and the two plinths attached to the rock abutments on the two sides (Figure 8a). First, these regions have been permanently covered by clean concrete faces since the first scanning epoch, while other nearby regions were constantly affected by seasonal vegetation changes and other disturbances. Furthermore, the intake area was particularly interspersed with a batch of well-spaced concrete anchors, which provided a unique shape feature for applying the ICP algorithm. Lastly, but most importantly, the dam foundation and abutment at the site are composed of relatively intact hard rock (see section of “Project overview”), which were expected to exhibit negligible deformation behavior during dam filling. Therefore, points within these three unvaried regions were used as the stable datums for geo-referencing five point clouds (in Table 1) into the same coordinate system. Note that only regions of interest (i.e., the upstream face and its nearby areas) were reserved during the geo-referencing process while others were cut out to avoid the accumulation errors. Afterwards, the “point-to-surface” based method was applied to the point clouds in the upstream face (e.g., extrusion wall area) to obtain its deformation behavior from one epoch to another. 

In order to elaborate the feasibility of the proposed strategy, the outcome of geo-referencing two stable datum regions acquired in epochs 1910 and 2007 is first illustrated herein (Figure 8b,c). The computed distances between these two epochs are unnoticeable (close to zero) over the majority of these regions, expect for those at the anchor rods (see Figure 8b). These local bulging spots of the anchor rods and their sporadic distribution provide unique benefits in terms of implementing the ICP algorithm, but this feature can also results in surface generation errors that is the main cause of the observable difference at these local spots. Nevertheless, this systematic difference is rather small and has no adverse effect on deformation results presented later. Figure 8d,e show the probability density of the calculated deviation (nominal deformation) for the intake area and plinths, respectively, which approximates to normal distributions and average deviation centers around zero, with 95% confidence intervals (CI) of [−15 mm, 15 mm] and 85% CI of [−10 mm, 10 mm]. Similar statistical results were obtained for the several subregions of these datum area, recalling that the errors can be resulted from multiple resources (e.g., intrinsic ranging errors and reflectivity deference) and also noting that surface generation errors (>15–20 mm) at those anchor rods account for a certain percentage. However, the fact that these errors center around zero supports the assumption that the selected areas remained stable over time. According to [38], the accuracy for displacement measurements should be <10 mm for embankment dams during construction with traditional geodetic instruments. These statistical deviation results in Figure 8 show that the proposed strategy can provide satisfactory accuracy for deformation evaluation if the surface generation errors are excluded. These ineluctable numerical errors are one or two orders of magnitude smaller that the deformation values over the upstream surface presented in the following section.

### 4.3. Results of Deformation Trends

**Figure 9:**
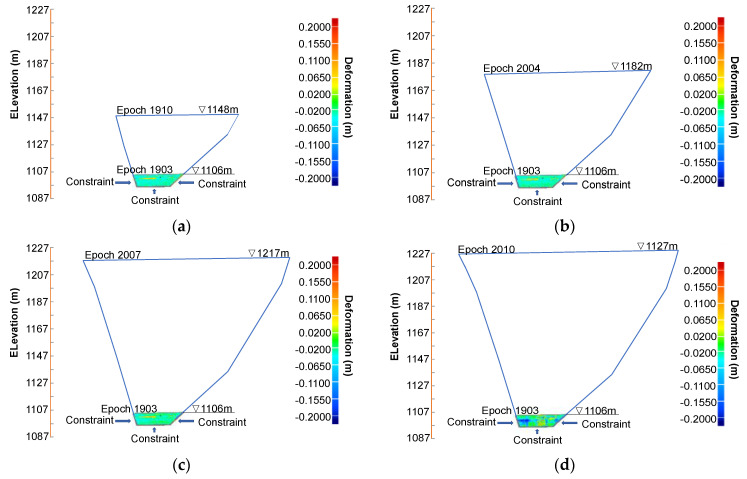
Three-dimensional displacement distribution over the extrusion wall with epoch 1903 as the reference data set: (**a**) epoch 1910 as test data; (**b**) epoch 2004 as test data; (**c**) epoch 2007 as test data; and (**d**) epoch 2010 as test data.

**Figure 10:**
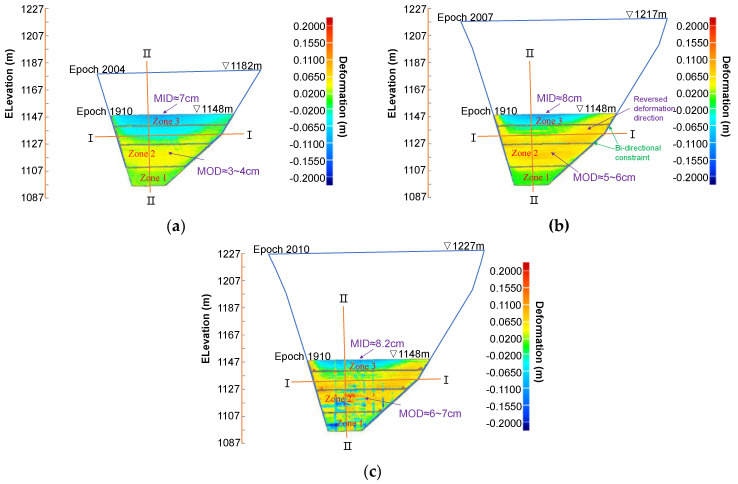
Three-dimensional displacement distribution over the extrusion wall with epoch 1910 as the reference data set: (**a**) epoch 2004 as test data; (**b**) epoch 2007 as test data; and (**c**) epoch 2010 as test data.

**Figure 11:**
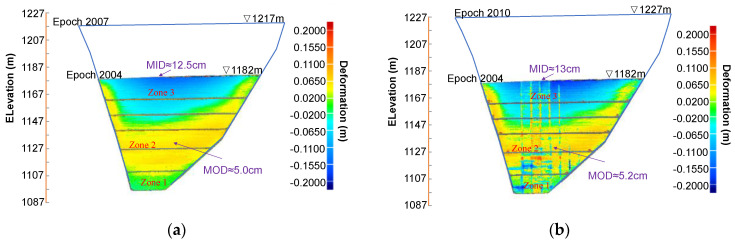
Three-dimensional displacement distribution over extrusion wall with the epoch 2004 as the reference data set: (**a**) epoch 2007 as test data; and (**b**) epoch 2010 as test data.

**Figure 12:**
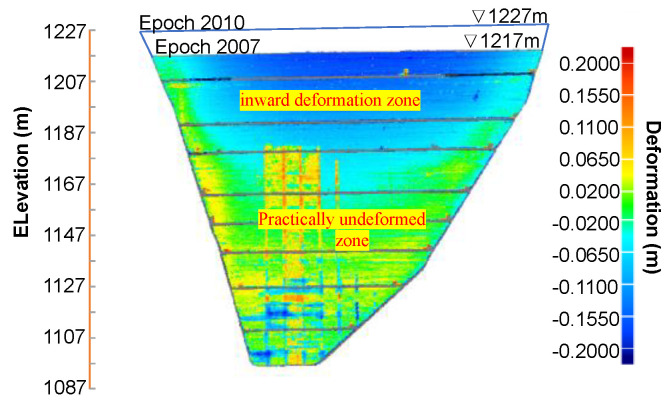
Three-dimensional displacement distribution over the extrusion wall with epoch 2007 as reference data set and epoch 2010 as test data.

Figure 9, Figure 10, Figure 11 and Figure 12 demonstrate the 3D displacement distributions over the extrusion wall that were calculated using the epochs of 1903, 1910, 2004 and 2007 as the reference data sets, respectively. The blue and red hues imply that the test epochs display an outward convex and inward concave deformation, respectively, compared to reference epochs. The area close to the dam base exhibited small inward deformations (around 3 cm) from epoch 1903 to 1910, which was induced by filling rock material right above. The deformations essentially stopped at some point before epoch 1910, as no deformation increments are observed at all subsequent epochs (2004, 2007 and 2010) relative to 1903 (Figure 9b–d). The consistent stabilization of this area is attributed to the well constraints from the firm base and two stiff sides, and its small narrow space certainly exaggerates constraint effects. 

For reference epoch 1910, deformation patterns can be divided into three zones over all three following periods (see Figure 10a–c). Zone 1 (bottom zone) is the stable zone (zero deformation) with a trapezoid geometry that follows the nature valley shape, for which its dimensions (≈12 m height) remain remarkably unchanged. Zone 2 (middle zone) has a half-moon shape with the concave boundary facing upwards, within which the intrusion side wall deforms toward the outside of the rockfill. The maximum outward deformation (MOD) is 3–4 cm and 5–6 cm for epochs 1910–2004 and 1910–2007, respectively, but it occurs consistently in the middle of this zone. Zone 3 (upper zone) has an imperfect and asymmetric arc shape, within which the intrusion side wall deforms toward the inside of the rockfill. The maximum inward deformation (MID) is 7 cm and 8 cm for epochs 1910–2004 and 1910–2007, respectively, but it occurs consistently at the upper boundary of this zone. The latter two zones are split by a zero-deformation band. This implies that the gradual filling of the rock will first induce a general settlement of the existing dam body right below the filling surface, and the continuous filling at the far top face tends to reverse the deformation direction in the relative lower zone due to the weight transmission along the sloping effect (see Figure 10b for example). As observed, the half-moon (crescent) shaped, outward deformation zone (zone 2) expands upwards and invades into the previously inward deformation zone (zone 3) from epoch 1910 to 2007, compared with epoch 1910 to 2004. The invasion is particularly remarkable along the two wings of the plinth due to its unidirectional constraints, forming the two corners of crescent. Its asymmetry is assuredly linked to the asymmetric valley shape (marked in Figure 10b, for instance). On the right side, the corner of the bi-liner shaped plinth offers a bi-directional constraint (marked in Figure 10b for instance) to deformation compared with the unidirectional constraint on the left. Thus, the magnitudes of outward deformation and its area are both larger on the right side. Moreover, the slightly gentler average slope on the right side also contributes to this asymmetric deformation distribution.

The 3D deformation pattern and its evolution after the reference epoch 2004 (Figure 11) resemble those with epoch 1910 as reference (Figure 10) with three distinct zones. MOD is about 5.0 cm for epoch 2004–2007 and occurs around the center of the middle zone (at elevation of 1130 m). MID is around 12.5 cm for epoch 2004–2007 and occurs at the upper boundary of the upper zone. It is worth mentioning that the deformation results for the two epochs 2004–2007 and 2004–2010 can also be obtained by subtracting the deformation for 1910–2004 from those for the two epochs 1910–2007 and 1910–2010. This will be helpful for a better interpretation of deformation pattern evolution with the 2D cross-section results, as discussed later.

When comparing the data of epochs 1910–2010 (Figure 10c) and 2004–2010 (Figure 11b) to the respective previous results (Figure 10b and Figure 11a), no significant extra deformations are observed after epoch 2007. One reason for this might be that the additional rock filling between epoch 2007 and 2010 was significantly lesser than previous intervals (around 10 m thick in Figure 6). Moreover, the existing underlying dam body is much thicker and wider and allows the full absorption of the settlement increment from epoch 2007 to 2010, as indicated in the upper inward deformation zone in Figure 12. The remaining zone below remains practically undeformed (marked in Figure 12). The outliers in the data set associated with epoch 2010 (see Figure 9d, Figure 10c, Figure 11b and Figure 12) might be caused by interference from pre-pouring of concrete face slab. Nevertheless, the pairwise comparisons of all the epoch sequences feverously promises a full-field analysis of the 3D deformation distribution over the upstream face and its temporal evolutions with increasing dam height.

Table 2 summaries key statistics of the deformation features for a better comparison across the all the epochs. Overall, the additional filling from 2004 to 2007 has resulted in an average inward deformation increase of 2 cm and a maximum outward deformation (MOD) increase of 1 cm, when comparing results for epoch 1910 as the reference. Similarly, these values from 2007 to 2010 were only 0.5 cm and 0.2 cm, respectively, for epoch 1910 as the reference. This indicates that addition filling far away is likely to have an insignificant influence on the deformation of the dam body at lower elevations. The inward deformations are consistently higher than the outward deformations for all epochs, and the difference increases as the volume of dam body increases, e.g., the inward deformation is more than 2 times that of outward deformation at the lower elevation for epoch 2004–2010. This is because the deformation can accumulate in a much larger volume of dam body. Thus, the maximum inward deformation reaches the largest value of 15 cm for epoch 2007–2010.

**Figure 13:**
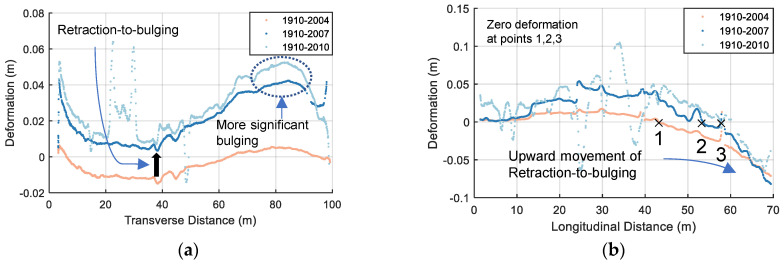
Deformation distribution along 2D cross sections with reference epoch of 1910: (**a**) I-I in the horizontal plane; and (**b**) II-II in the vertical plane.

**Figure 14:**
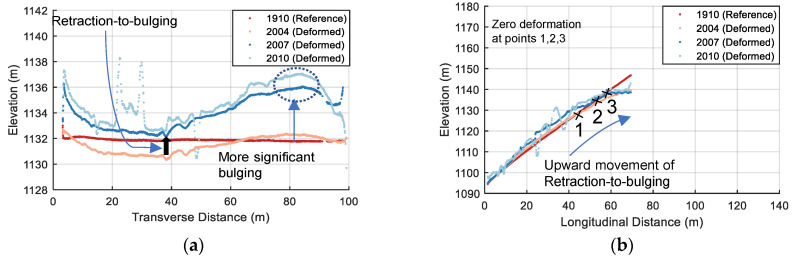
Two-dimensional deformed shape at three epochs 2004, 2007 and 2010 compared with reference epoch 1910 (deformation magnitudes are magnified 100 times) at cross sections: (**a**) I-I; and (**b**) II-II.

Likewise, the data from any 2D cross section can be singled out either for special spot checks or for more detailed information regarding to the evolutive deformation pattern. For example, Figure 13 shows the deformation distribution along cross-sections I-I and II-II in the horizontal (transverse) and vertical (longitudinal) plane, respectively, with epoch 1910 as the reference data. The locations of these two cross-sections are shown in Figure 10. It is clearly observed that a concave inward (negative) deformation distribution along cross-section I-I for epoch 1910–2004 has been reversed to a convex bulging (positive) deformation for epoch 1910–2007, which occurs first near the plinth and spreads to the middle (see Figure 13a). While the upward movement of the retraction-to-bulging phenomenon is displayed in Figure 13b (e.g., zero deformation moves from Point 1 to 3). Moreover, the asymmetric deformation behaviour (i.e., more significant bulging near the left abutment) is also observed. According to the calculated results, the deformed shape of the extraction wall at the corresponding cross sections can be better viewed by magnifying the deformation values. Figure 14 shows the deformed shape at these two cross-sections at three epochs 2004, 2007 and 2010. The deformation magnitudes are magnified 100 times compared with the reference epoch 1910. The deformation behaviours discussed above (e.g., retraction-to-bulging) can be more intuitively appreciated in these plots.

## 5. Conclusions

In the realm of construction of large-scale infrastructures (e.g., concrete face rock dams or CFRDs), the advanced cost-efficient non-contact monitoring technique and effective methods for processing the associated large-scale data are in high demand for the goal of building intellectualization. Within this paper, we presented an application of the terrestrial laser scanning (TLS) for inventory of the as-is shape of a 183-m high CFRD at six different epochs over two years of construction. Our field work showed that a well-proportioned geodetic system is the fundamental key for capturing, in a well-ordered manner, the massive dam’s panorama in the narrow valley and for facilitating data postprocessing of geo-registering and geo-referencing. Several general principles were suggested for selecting permanent scanning positions considering that a specific system should be dependent on site conditions.

More emphasis was placed on extracting the 3D deformation spatial distribution and its temporal evolution over the dam’s upstream face, which was achieved by exploiting high data redundancy with a tailored point cloud processing pipeline. A critical step is to select undeformed/unchanged zones with featured geometrical shape as the datum for aligning the multiple point clouds in a common system. Extensive statistical study revealed the high accuracy of this local datum-based alignment, and the nominal deformations over datums showed normal distributions and centered at zero. The minimum detectable deformation is in the range of below ±10.0 mm if the numerical errors of surface generation were removed. These values meet the accuracy requirement suggested in the literature [38] based on traditional highly precise, point-wise surveying methods.

However, the proposed monitoring scheme stands out in terms of providing a complete picture of the spatio-temporal evolution of the dam’s deformation behaviour. Several important deformation characteristics, which could never be obtained by the traditional methods, were obtained in this study in an efficient and cheaper manner. Three deformation zones from the valley bottom to the dam top were distinguished during the dam construction process, namely, a practically undeformed zone, a bulging deformation zone and a retraction deformation zone. The unformed zone is limited to a certain dimension due to the well constraints from the narrow and stiff valley base. The retraction (inward deformation) zone near the top is due to the general settlement induced by the filling weight. The previous retraction zone will evolve to the bulging zone with outward deformation due to the weight transmission along the sloping effect with continuous filling at the far top face. The results clearly demonstrate the upward trajectory of the zero-deformation band that separates retraction and bulging zones. Furthermore, typical asymmetrical valley effects were also well indicated by different upward rates of retraction-to-bulging at two dam abutments in the analysis.

The present monitoring strategy provides abundant objective data for tracking the deformation behaviour of dam that is being constructed in a narrow, asymmetrical valley. Its widespread application at high frequency (more intensive epochs) is promising for optimizing the construction schedule and for refining the numerical models that are commonly used by experts to foretell the dam behaviour in detail. This study provides a good and initial reference for standardizing field monitoring work and provides accurate data interpretation procedures for these purposes.

## Figures and Tables

**Table 1:** Pairwise comparison for deformation calculation.

Pairwise Comparison	Test (Points)			
References (Surface)	31 October 2019	25 April 2020	29 July 2020	15 October 2020
17 March 2019	√ ^1^	√	√	√
31 October 2019	N.A. ^2^	√	√	√
25 April 2020	N.A.	N.A.	√	√
29 July 2020	N.A.	N.A.	N.A.	√

Note: ^1^ √ represents that the deformation analysis is performed; ^2^ N.A. represents that the deformation analysis is not required.

**Table 2:** Key statistics of the deformation features.

Epoch		Filling Thickness (m)	Deformation			
Reference	Test		Direction	Elevation Range (m)	Maximum Value (cm)	Maximum Value Elevation (m)
1910	2004	31	Inward	1127–1148 (20)	7	1148
			Outward	1107–1127 (20)	3–4	1117
	2007	70	Inward	1137–1148 (10)	8	1148
			Outward	1107–1132 (30)	5–6	1117
	2010	81	Inward	1140–1148 (8)	8.2	1148
			Outward	1107–1136 (30)	6–7	1120
2004	2007	39	Inward	1141–1182 (40)	12.5	1182
			Outward	1107–1139 (30)	5	1130
	2010	50	Inward	1141–1182 (40)	13	1182
			Outward	1107–1139 (30)	5.2	1130
2007	2010	11	Inward	1152–1217 (65)	15	1217
			Outward	/	/	/

## Data Availability

Not applicable.
